# ρ^0^ Cells Feature De-Ubiquitination of SLC Transporters and Increased Levels and Fluxes of Amino Acids

**DOI:** 10.3390/ijms18040879

**Published:** 2017-04-20

**Authors:** André Bordinassi Medina, Marcin Banaszczak, Yang Ni, Ina Aretz, David Meierhofer

**Affiliations:** 1 Max Planck Institute for Molecular Genetics, Ihnestraße 63-73, 14195 Berlin, Germany; andrebmedina@gmail.com (A.B.M.); ni@molgen.mpg.de (Y.N.), ina.aretz@gmx.de (I.A.); 2 Department of Biochemistry and Human Nutrition, Pomeranian Medical University, Broniewskiego 24, 71-460 Szczecin, Poland; banaszczak.marcin@gmail.com

**Keywords:** SLC transporter, ubiquitination, amino acids, pulse chase, ρ^0^ cells

## Abstract

Solute carrier (SLC) transporters are a diverse group of membrane transporter proteins that regulate the cellular flux and distribution of endogenous and xenobiotic compounds. Post-translational modifications (PTMs), such as ubiquitination, have recently emerged as one of the major regulatory mechanisms in protein function and localization. Previously, we showed that SLC amino acid transporters were on average 6-fold de-ubiquitinated and increased amino acid levels were detected in ρ^0^ cells (lacking mitochondrial DNA, mtDNA) compared to parental cells. Here, we elucidated the altered functionality of SLC transporters and their dynamic ubiquitination status by measuring the uptake of several isotopically labeled amino acids in both human osteosarcoma 143B.TK- and ρ^0^ cells. Our pulse chase analysis indicated that de-ubiquitinated amino acid transporters in ρ^0^ cells were accompanied by an increased transport rate, which leads to higher levels of amino acids in the cell. Finding SLC transport enhancers is an aim of the pharmaceutical industry in order to compensate for loss of function mutations in these genes. Thus, the ubiquitination status of SLC transporters could be an indicator for their functionality, but evidence for a direct connection between de-ubiquitination and transporter activity has to be further elucidated.

## 1. Introduction

Solute carrier (SLC) transporters, classified into 52 families with about 400 members in total, are a group of integral transmembrane proteins that facilitate the transport of substrates and ions across biological membranes [1]. SLC transporters are located in various membranes, such as plasma-, lysosomal-, inner mitochondrial-, peroxisomal membrane, and endoplasmic reticulum in an organ or cell type specific manner. Several SLC transporters have been identified as tumor suppressors [2,3,4,5] and are of special interest in drug pharmacokinetics, as they can be used as drug targets to modulate the transportation of small molecules [1,6,7,8]. For example, SLC1A5 (also known as ASCT2), a neutral amino acids transporter, was shown to be a potential therapeutic target for melanoma, as the transporter activity could be reduced by a specific inhibitor and thereby reduce the amount of amino acid uptake and subsequently cell proliferation and cell cycle progression [9]. Furthermore, 20% of all known SLC transporters have been associated with Mendelian disease with a wide range of symptoms, as reviewed in Lin et al. [1]. Current drugs are addressing transporter defects by inhibiting their function, but most gene defects actually result in a loss of function. Thus, a functional enhancement of defective SLC transporters is needed to compensate for the gene defect. So far, only the drug riluzole was shown to be a SLC transport enhancer for the transporters SLC1A1, SLC1A2, and SLC1A3 [10,11,12,13].

Ubiquitination is one of the most common post-translational modifications. Target proteins for ubiquitination are modified by the covalent attachment of either a single ubiquitin (mono-ubiquitination) or a chain of ubiquitins (poly-ubiquitination). This process can be reversed by de-ubiquitinases (DUBs). All seven lysine residues of ubiquitin as well as its N-terminus can serve as points of ubiquitination. As an example, lysine 48-linked poly-ubiquitin chains target proteins to the 26S proteasome for degradation, whereas the 63-linkage type is involved in processes such as endocytic trafficking, inflammation, translation, and DNA repair. Thus, the linkage type determines the fate of target proteins [14]. Recently identified mixed and branched ubiquitin chain linkage types are just starting to be decoded [14,15,16,17]. New methods, for instance, an “ubiquitin interactor affinity enrichment-mass spectrometry” approach in proteome-wide analyses, have been applied to decipher the complex language of ubiquitin signaling [18].

The ubiquitination system plays an important role in cancer, as about 800 E3 ubiquitin ligases and 50 DUBs tune protein abundancies, which are frequently altered in cancer. For example, E3 ubiquitin ligases dominantly regulate protein levels and activities of the tumor suppressor TP53 and are therefore considered to be a new class of biomarkers and therapeutic targets in diverse types of cancers [19].

Besides serving as building blocks for protein synthesis, selective amino acids are precursors for the synthesis of purines and pyrimidines, glutathione, and epigenetic marks, and can activate the mTOR complex 1 (mTORC1) pathway. The proper supply of amino acids to all tissues and the homeostasis of plasma amino acid levels are therefore critical. The deficiency of SLC transporters can cause severe inborn disorders of amino acid transportation [20].

Previously, we integrated the data of proteome and metabolome profiling of the human parental osteosarcoma cell line 143B.TK- versus cells lacking mtDNA (ρ^0^ cells) [21]. We identified an unexpected de-ubiquitination of all identified SLC amino acid transporters as well as an increased level of amino acids in ρ^0^ cells [21]. This integrative proteomics and metabolomics study raised the question of whether or not the detected 6-fold de-ubiquitination of SLC amino acid transporters in ρ^0^ cells correlates to fluxes and influences the activity of the SLC transporters, as the total amount of SLC transporters was unchanged. Therefore, we monitored the absolute and relative amount of 16 unlabeled and isotopically labeled (^12^C^14^N and ^13^C^15^N) amino acids in pulse chase approaches in both cell lines by applying a targeted liquid chromatography mass spectrometry (LC-MS) profiling method, based on multiple reaction monitoring (MRM). The results enabled us to shed light on differences in amino acid transportation kinetics, depending on the ubiquitination status of solute carrier transporters between ρ^0^ cells and the parental cell line 143B.TK-.

## 2. Results and Discussion

**Figure 1:**
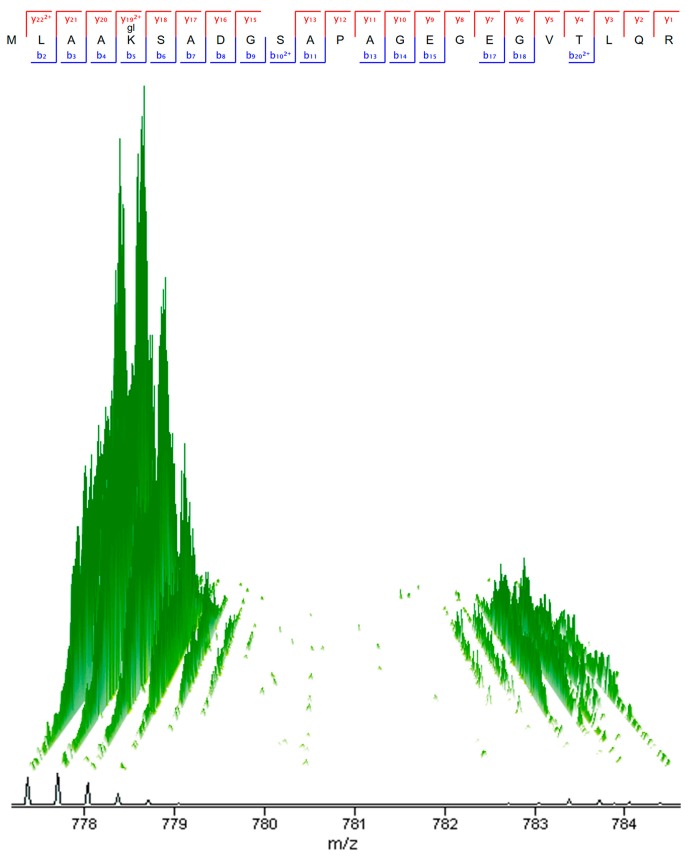
A stable isotope labeling with amino acids in cell culture (SILAC) pair of an ubiquitinated solute carrier (SLC) transporter peptide. Isotopic patterns (^13^C) of the precursor mass spectrometry (MS1) peaks of the peptides (MLAAK_(Ubi)_SADGSAPAGEGEGVTLQR, *m*/*z* 777.72 and 783.72, charge 3^+^, MS score 195.36) from SLC7A5 are displayed in three dimensions (3D) from the SILAC pairs of unlabeled 143B.TK- (peaks on the left) and ^13^C^15^N labeled ρ^0^ cells (peaks on the right). Labelled lysine (Lys8) and arginine (Arg10) in ρ^0^ resulted in a mass shift of 6 Da. The identified MS^2^ y- and b ion series of the peptide is indicated above the 3D peaks. The 32-fold peak volume decrease of the heavy peak indicates the tremendous de-ubiquitination in ρ^0^ cells.

Our previous proteome analysis identified a severe de-ubiquitination of SLC transporters in ρ^0^ cells. The 44 quantified ubiquitinated SLC transporter peptides showed an average of only one sixth of ubiquitin left in ρ^0^ cells compared to 143B.TK- cells (Table S1) [21]. A three dimensional (3D) peak of a stable isotope labeling with amino acids in cell culture (SILAC) peptide pair of an ubiquitinated SLC transporter protein is displayed in Figure 1 to highlight the difference between the ubiquitin status of an exemplary transporter in ρ^0^ and 143B.TK- cells.

### 2.1. Amino Acid Flux of ρ^0^ and 143B.TK- Cells

**Figure 2:**
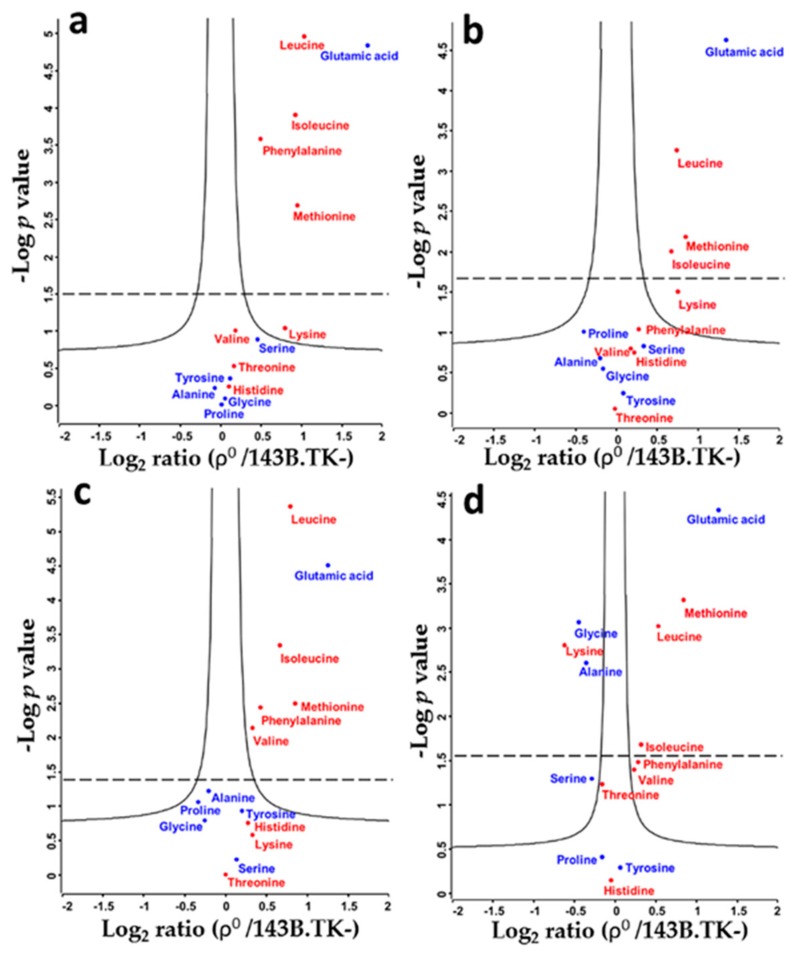
Volcano plots of relative amino acid levels between ρ^0^ and 143B.TK- cells after switching the culture medium from unlabeled to labeled amino acids at different time points. Shown are ^13^C^15^N amino acid ratios at (**a**) 2.5 min, (**b**) 5 min, (**c**) 10 min, and (**d**) 20 min. Significantly altered amino acids are above the continuous line and in addition after Benjamini-Hochberg (BH) correction above the dashed line. Essential amino acids are in red.

We applied a targeted LC-MS methodology to identify and quantify relative differences in intracellular amino acid levels between de-ubiquitinated ρ^0^ and parental 143B.TK- cells. Except arginine and aspartic acid, all monitored amino acids were detected and relatively quantified. Ratios of ^13^C^15^N labeled amino acids were displayed in volcano plots for the time points 2.5, 5, 10, and 20 min after the medium swap from unlabeled to labeled amino acids in ρ^0^ versus 143B.TK- cells (Figure 2). We observed an average 1.45-fold up-regulation of essential and 1.2-fold up-regulation of non-essential amino acids already within 2.5 min (Figure 2a) after the label swap in the ρ^0^ state. None of the detected amino acids at this time point were downregulated. Similar regulations were observed at time points 5 and 10 min (Figure 2b,c). Several amino acids showed a significantly higher amount in the ρ^0^ state at all time points, such as methionine, isoleucine, leucine, and glutamic acid. Interestingly, all significantly upregulated amino acids in ρ^0^ cells, except glutamic acid, were essential amino acids.

Only after 20 min, several amino acids were significantly downregulated in ρ^0^ cells, such as glycine, lysine, and alanine (Figure 2d). The entire list of integrated and normalized peak areas for all six biological replicates is given in Table S2.

**Figure 3:**
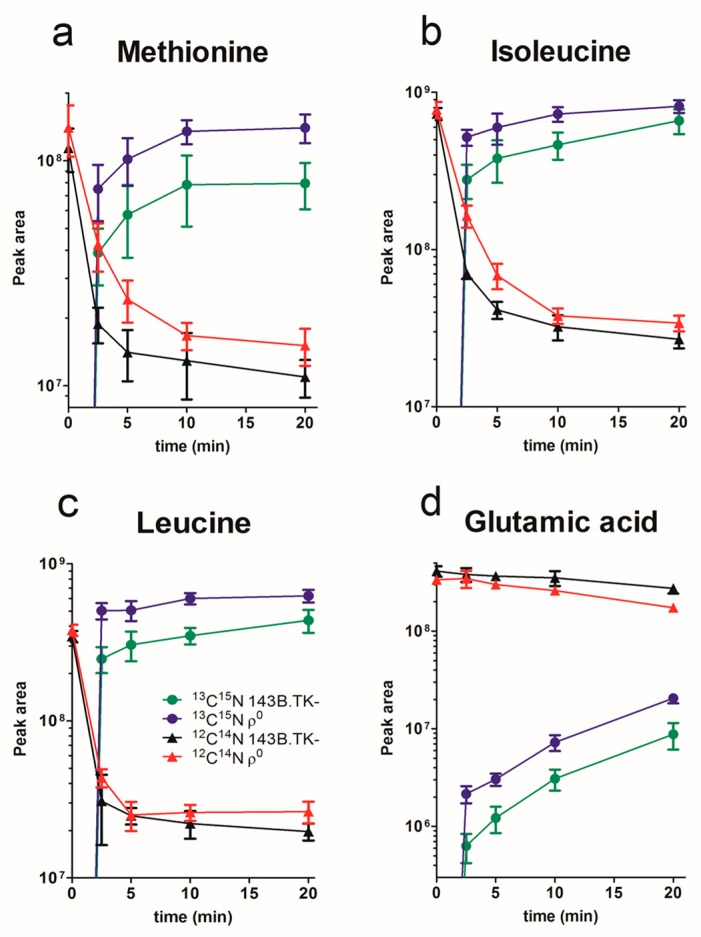
Decrease and increase of significantly regulated amino acids after switching the culture medium from unlabeled to labeled amino acids in ρ^0^ and 143B.TK- cells (log10 scale). The peak areas (in counts per second) are shown for (**a**) methionine, (**b**) isoleucine, (**c**) leucine, and (**d**) glutamic acid. ^13^C^15^N amino acids of 143B.TK- cells are shown in in green circles, ^13^C^15^N amino acids of ρ^0^ cells in blue circles, ^12^C^14^N amino acids of 143B.TK- cells in black triangles, and ^12^C^14^N amino acids of ρ^0^ cells in red triangles. Data were expressed as mean and standard deviation (mean ± SD; *n* = 6).

To display the relationship between the decrease of unlabeled and the increase of labeled amino acids between ρ^0^ and 143B.TK- cells, we generated a time series plot of amino acids with significantly regulated levels at all time points (Figure 3).

The ^13^C^15^N labeled essential amino acids methionine, isoleucine, and leucine reached endogenous concentrations already after a few minutes, and the concentration was always higher in ρ^0^ cells. The uptake speed of ^13^C^15^N labeled glutamic acid was in contrast much slower and did not reach the plateau after 20 min. One reason might be that non-essential glutamic acid, a key compound in cellular metabolism, is synthesized by transamination of alanine and aspartate. This metabolic pathway had to be used before the label switch by the cells, because the ^12^C^14^N cell culture media was not supplemented with glutamic acid (Table S3). In comparison, ^13^C^15^N glutamic acid was supplemented in the labeled media, adoption of metabolic pathways and transportation via SLC transporters had therefore to be established first. The synthesis from other compounds is a much slower process than the import of metabolites, as such, the saturation plateau will therefore be reached later. Furthermore, the “old stock” of unlabeled precursor amino acids will be used in congruence with newly imported ^13^C^15^N glutamic acid. The increase of labeled compounds to calculate the flux dynamics of amino acids by SLC transporters can ergo only be monitored in the log phase, as it is not possible to distinguish between the uptake by transporters or the conversion of amino acids at a later stage.

Our data indicate that the uptake and saturation level of intracellular isotopically labeled amino acids to its final concentration is completed within minutes for essential amino acids. Non-essential amino acids are interconvertible, products and educts of many other cell processes influence the intracellular pool of amino acids and prolong the time to reach the plateau.

Many SLC transporters are not exclusively responsive for one compound, rather transporting a wide range or group of compounds (e.g., neutral-, anionic-, and cationic amino acids) with different efficiencies depending on physiological conditions [22]. Thus, this pulse chase experiment indicates a significant altered transportation rate of several amino acids in ρ^0^ cells.

### 2.2. Determination of Absolute Amino Acid Concentrations

**Figure 4:**
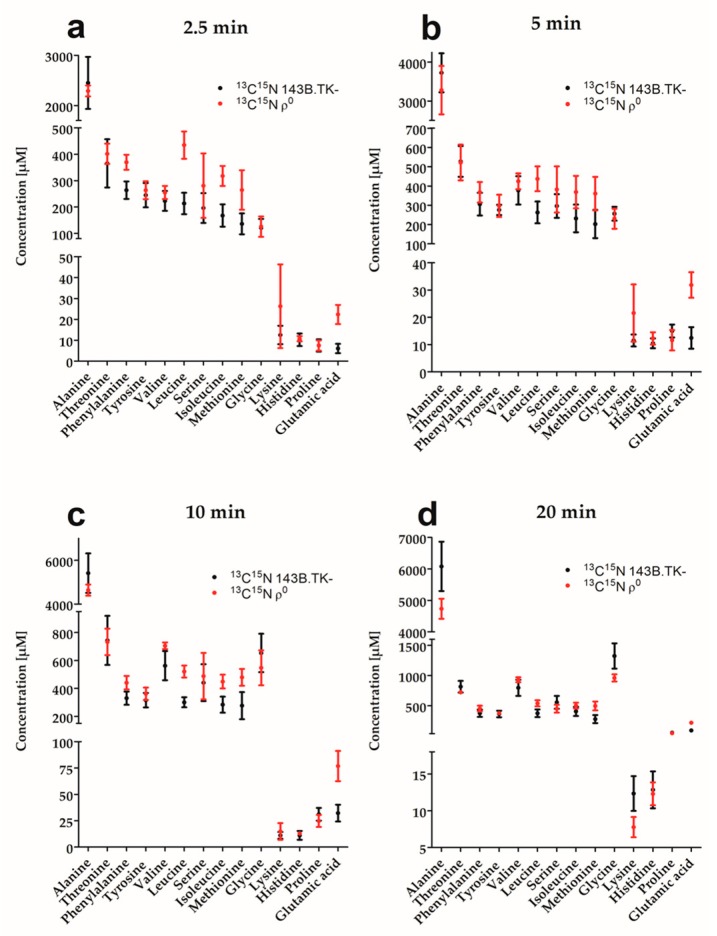
Absolute concentration of labeled amino acids for ρ^0^ (red) and 143B.TK- (black) cells after switching the culture medium from unlabeled to labeled amino acids. Concentrations for ^13^C^15^N amino acid are shown in µM at time points: (**a**) 2.5 min; (**b**) 5 min; (**c**) 10 min; and (**d**) 20 min. Data were expressed as mean and standard deviation (mean ± SD; *n* = 6).

A standard curve of ^13^C^15^N amino acids was used to calculate and compare absolute amounts of labeled amino acids in ρ^0^ and 143B.TK- cells after each time point (Figure 4).

For almost all amino acids and time points, we measured a higher absolute concentration of ^13^C^15^N amino acid in ρ^0^ cells. As the concentration of labeled and unlabeled amino acids in the culture medium varied up to 10-fold (Table S3), the final absolute amino acid concentration differed between the labels and was only compared between cell lines of the same label, but not between labels. Higher amino acid fluxes as well as absolute concentrations indicate a general higher demand on amino acids in ρ^0^ cells.

### 2.3. Solute Carrier Transporter Inhibition

**Figure 5:**
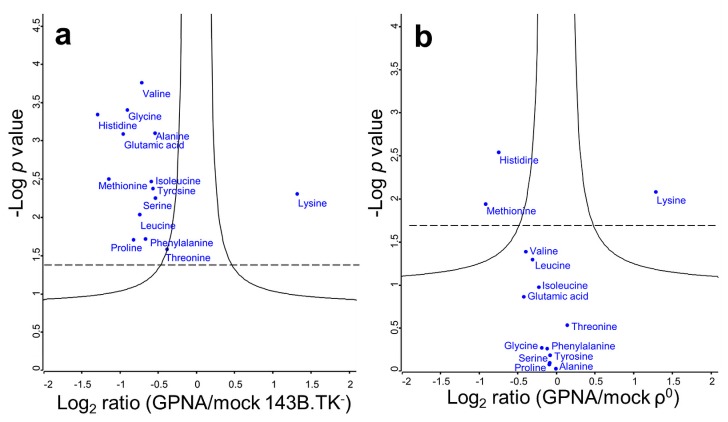
The SLC transporter inhibitor γ-glutamyl-*p*-nitroanilide (GPNA) significantly decreased the amount of almost all ^13^C^15^N amino acids in (**a**) 143B.TK- cells, but had no effect on (**b**) ρ^0^ cells. Significantly altered amino acids were above the continuous curve and in addition after Benjamini-Hochberg correction above the dashed line.

γ-glutamyl-*p*-nitroanilide (GPNA, a synthetic glutamine analogue) was discovered to be a specific SLC1A5 inhibitor [23]. We used this inhibitor to elucidate which proportion of the amino acid flux is specific to this neutral amino acid transporter. Glutamic acid, alanine, serine, cysteine, and glutamine were previously shown by Esslinger et al. [23] to be the main affected amino acids by GPNA inhibition (range: 50–100%). Except lysine, all amino acids were significantly reduced after 10 min in 143B.TK- cells (Figure 5a, Table S4), indicating a potent and wide range inhibition of the SLC transporter. On average, amino acids were decreased by 1.6-fold. Many SLC transporters have a broad range of selectivity; the inhibition of one transporter just slightly reduces the total flux, as other transporters can compensate. In contrast, GPNA failed to inhibit de-ubiquitinated SLC transporters in ρ^0^ cells, as only a 1.09-fold reduction was observed (Figure 5b, Table S4). The essential amino acid lysine was the only significantly increased ^13^C^15^N amino acid in both cell types after GPNA treatment; histidine and methionine were downregulated in both ρ^0^ and parental cells.

New data indicate that GPNA indeed inhibits not only the Na^+^-dependent influx, but also affects the Na^+^-independent uptake, and thus is also an inhibitor for SLC7A5 [24]. Why can ρ^0^ cells exclusively bypass inhibition by GPNA? We assume that the ubiquitination status has little, if any, influence, as typically only much less than 1% of a protein is ubiquitinated [25]. Therefore, we speculate that the microenvironment compensates or influences the inhibitor binding behavior. It is an interesting observation per se that ρ^0^ cells revoke the effectiveness of GPNA and might be connected to the energy status, the main difference between ρ^0^ cells and 143B.TK- cells.

Oxidative phosphorylation is the most efficient way to convert nutrition substrates such as carbohydrates, amino acids, and fatty acids into adenosine triphosphate (ATP). The only genetic difference in ρ^0^ cells compared to their parental cells is the lack of mtDNA, resulting in an incomplete and non-functional oxidative phosphorylation system. Glycolysis produces only 2 ATP molecules, oxidative phosphorylation between 30 and 36 ATPs. In order to generate the same amount of ATP in ρ^0^ cells, a higher uptake of according metabolites is necessary. A similar situation is present in most cancers, running their metabolism under aerobic glycolysis with a high nutritional demand to support their rapid growth. As a result, many glucose [26] and lactic acid [27] transporters are therefore upregulated in cancer. To address this higher need of amino acids, de-ubiquitination of SLC transporters could facilitate this demand without changing the expression profile. Furthermore, detachment of ubiquitin could change the conformation or localization of the SLC transporter in order to modulate the flux. However, these are very unlikely scenarios, as the average stoichiometry of this modification is low (below 1%) [25]. Therefore, de-ubiquitination of ρ^0^ cells is unlikely the sole reason for the enhanced SLC transporter activity. More likely, the Vmax of SLC transports did not reach the maximum in both cell lines yet and the observed differences are due to distinct energy demands. In addition, ρ^0^ cells have no functional respiratory chain, as such, the main source of reactive oxygen species (ROS) were about 90% of ROS are normally generated is absent. Hence, the need to degrade defective oxidized transporters or proteins through the 26S proteasome via ubiquitination is reduced. This is supported by the global de-ubiquitination found in ρ^0^ cells [21]. The molecular mechanism for the enhanced transporter activity in ρ^0^ cells still remains to be discovered.

## 3. Methods

### 3.1. Cell Culture

A thymidine kinase deficient (TK-) osteosarcoma cell line 143B.TK- (ATCC-CRL-8303) with bromodeoxyuridine resistance was obtained from LGC Standards (Wesel, Germany) and is the parental line of ρ^0^ cells, following the protocol from [28]. The wild-type cell line 143B.TK- and the according ρ^0^ cells were cultivated in Dulbecco’s Modified Eagle Medium (DMEM, Silantes, Munich, Germany), containing 4.5 g/L glucose, 1 mM pyruvate, supplemented with 5% fetal bovine serum (Sigma-Aldrich Munich, Germany), 1% Penicillin-Streptomycin-Neomycin (Invitrogen, Carlsbad, CA, USA), 100 µg/mL bromodeoxyuridine (Sigma-Aldrich), and 50 µg/mL uridine (Sigma-Aldrich) at 37 °C in a humidified atmosphere of 5% CO_2_. Cells were seeded half-confluent into 9.2 cm^2^ polystyrene plates in at least triplicates for every condition.

### 3.2. Amino Acid Flux Assay

In order to elucidate quantitative differences in amino acid fluxes between 143B.TK- and ρ^0^ cells by SLC transporters, both cell lines were grown in 9.2 cm^2^ culture plates for 24 h to reach confluence in biological sextuplicates. DMEM medium was replaced 4 h before the experiment to guarantee equal starting conditions and to avoid nutrient depletion and acidification. Cells were then washed twice in 1× phosphate-buffered saline (PBS), pH 7.4, and incubated for 2.5, 5, 10, and 20 min in the following medium, respectively: 1 mL of Roswell Park Memorial Institute (RPMI) 1640 medium modified (US Biological, Salem, MA, USA) without amino acids and glucose, supplemented with 5% dialyzed FBS (Silantes), 1% Penicillin-Streptomycin-Neomycin (Invitrogen), 100 µg/mL bromodeoxyuridine (Sigma-Aldrich), 50 µg/mL uridine (Sigma-Aldrich), 1 mM pyruvate, sodium bicarbonate, 16 µg/mL tryptophan, an algal amino acids mixture containing 17 isotopically labeled amino acids (U-13C, 97%–99% and U-15N, 97%–99%, Cambridge Isotope Laboratories, Andover, MA, USA), and d-glucose (U-13C, 99%, Cambridge Isotope Laboratories). The concentration of culture media with labeled and unlabeled amino acids can be found in Table S3. The cells were harvested by two freeze and thaw cycles in liquid nitrogen, and the lysate was collected for metabolite extraction.

### 3.3. SLC Transporter Inhibitor Assay

The SLC transporter inhibitor GPNA, a potent commercially available competitive inhibitor of the SLC1A5 (ASCT2) transporter [23], was used to study which amino acids are indeed transported by SLC1A5.

Again, both cell lines were grown in 9.2 cm^2^ culture plates for 24 h to reach confluence, but with a pH lowered to 6, to reach optimal inhibiting effects. For each cell line, a control was incubated with isotopically labeled RPMI 1640 medium for 10 min as biological triplicates. For the inhibitor assay, cells were pre-treated with 1 mM GPNA for 10 min in DMEM, followed by a 1 mM GPNA incubation in isotopically labeled RPMI 1640 medium for another 10 min. Cells were treated identically for metabolite extraction as for the flux assay.

### 3.4. Metabolite Extraction and Profiling by Targeted Liquid Chromatography Mass Spectrometry (LC-MS)

Metabolite extraction and tandem LC-MS measurements were done as previously reported by us [29]. In brief, methyl-tert-butyl ester (MTBE), methanol, ammonium acetate, and water were used for metabolite extraction. Dry residuals were suspended in 25 µL acetonitrile and 25 µL methanol, centrifuged at 21,100× *g* for 5 min at 4 °C. The supernatants were transferred to microvolume inserts, 5 µL per run were injected for LC-MS analysis. Subsequent separation was performed on a LC instrument (1290 series UHPLC; Agilent, Santa Clara, CA, USA), online coupled to a triple quadrupole hybrid ion trap mass spectrometer QTrap 6500 (Sciex, Foster City, CA, USA), as reported previously [30]. Transition settings for multiple reaction monitoring (MRM) are provided in Table S5. All original LC-MS generated QTrap wiff files can be downloaded via http://www.peptideatlas.org/PASS/PASS00936.

The metabolite identification was based on three levels: (i) The correct retention time, (ii) up to three MRMs, (iii) and a matching MRM ion ratio of tuned pure metabolites as a reference [30]. Peak integration was performed using MultiQuant^TM^ software v.2.1.1 (Sciex). The integration setting was a peak splitting factor of 2, all peaks were reviewed manually and adjusted if necessary. The peak area of the first transition per metabolite was used for calculations, excluding pseudo transitions. Normalization was done according to total protein concentration by bicinchoninic acid (BCA) assay (Sigma-Aldrich) from aliquots of cell lysate and subsequently by the internal standard l-valine-d8 (25 µM final concentration). For absolute quantification, the first transition (excluding pseudo transitions) of according amino acids dilution series from the algal mix was used for the standard curves.

### 3.5. Statistical Analyses

Statistical analysis by a two-sample *t*-test with Benjamini-Hochberg (BH, false discovery rate (FDR) of 0.05) correction for multiple testing was applied. Data analyses were performed using Perseus (v1.5.3.2) [31], and Prism (GraphPad, La Jolla, CA). Data were expressed as mean and standard deviation (mean ± SD).

## 4. Conclusions

In summary, de-ubiquitinated SLC transporters showed a significantly higher flux of amino acids, resulting in higher intracellular amino acid levels in ρ^0^ versus 143B.TK- cells, but a direct link that de-ubiquitination is causative for the increased SLC transporter activity cannot be made. Many SLC transporters can be specifically inhibited, but only a very few can be activated, which would be of importance to treat inborn diseases of mutated transporter genes.

## Supplementary Materials

Supplementary materials can be found at www.mdpi.com/1422-0067/18/4/879/s1.

## Abbreviations

ATP	Adenosine triphosphate
LC-MS	Liquid chromatography mass spectrometry
MRM	Multiple reaction monitoring
SILAC	Stable isotope labeling with amino acids in cell culture
SLC	Solute carrier
PTM	Post-translational modification

## References

[1] Lin L., Yee S.W., Kim R.B., Giacomini K.M. (2015). SLC transporters as therapeutic targets: Emerging opportunities. Nat. Rev. Drug Discov..

[2] Bhutia Y.D., Babu E., Ramachandran S., Yang S., Thangaraju M., Ganapathy V. (2016). SLC transporters as a novel class of tumour suppressors: Identity, function and molecular mechanisms. Biochem. J..

[3] Li H., Myeroff L., Smiraglia D., Romero M.F., Pretlow T.P., Kasturi L., Lutterbaugh J., Rerko R.M., Casey G., Issa J.P. (2003). SLC5A8, a sodium transporter, is a tumor suppressor gene silenced by methylation in human colon aberrant crypt foci and cancers. Proc. Natl. Acad. Sci. USA.

[4] Schweinfest C.W., Henderson K.W., Suster S., Kondoh N., Papas T.S. (1993). Identification of a colon mucosa gene that is down-regulated in colon adenomas and adenocarcinomas. Proc. Natl. Acad. Sci. USA.

[5] Costello L.C., Franklin R.B. (2006). The clinical relevance of the metabolism of prostate cancer; zinc and tumor suppression: Connecting the dots. Mol. Cancer.

[6] Hopkins A.L., Groom C.R. (2002). The druggable genome. Nat. Rev. Drug Discov..

[7] Polillo M., Galimberti S., Barate C., Petrini M., Danesi R., Di Paolo A. (2015). Pharmacogenetics of BCR/ABL inhibitors in chronic myeloid leukemia. Int. J. Mol. Sci..

[8] Franke R.M., Gardner E.R., Sparreboom A. (2010). Pharmacogenetics of drug transporters. Curr. Pharm. Des..

[9] Wang Q., Beaumont K.A., Otte N.J., Font J., Bailey C.G., van Geldermalsen M., Sharp D.M., Tiffen J.C., Ryan R.M., Jormakka M. (2014). Targeting glutamine transport to suppress melanoma cell growth. Int. J. Cancer.

[10] Dall’Igna O.P., Bobermin L.D., Souza D.O., Quincozes-Santos A. (2013). Riluzole increases glutamate uptake by cultured c6 astroglial cells. Int. J. Dev. Neurosci..

[11] Daniel B., Green O., Viskind O., Gruzman A. (2013). Riluzole increases the rate of glucose transport in L6 myotubes and NSC-34 motor neuron-like cells via AMPK pathway activation. Amyotroph. Lateral Scler. Frontotemporal Degener..

[12] Fumagalli E., Funicello M., Rauen T., Gobbi M., Mennini T. (2008). Riluzole enhances the activity of glutamate transporters GLAST, GLT1 and EAAC1. Eur. J. Pharmacol..

[13] Carbone M., Duty S., Rattray M. (2012). Riluzole elevates GLT-1 activity and levels in striatal astrocytes. Neurochem. Int..

[14] Komander D., Rape M. (2012). The ubiquitin code. Annu. Rev. Biochem..

[15] Yau R., Rape M. (2016). The increasing complexity of the ubiquitin code. Nat. Cell Biol..

[16] Rajalingam K., Dikic I. (2016). Expanding the ubiquitin code. Cell.

[17] Herhaus L., Dikic I. (2015). Expanding the ubiquitin code through post-translational modification. EMBO Rep..

[18] Zhang X., Smits A.H., van Tilburg G.B., Jansen P.W., Makowski M.M., Ovaa H., Vermeulen M. (2017). An interaction landscape of ubiquitin signaling. Mol. Cell.

[19] Sane S., Rezvani K. (2017). Essential roles of E3 ubiquitin ligases in p53 regulation. Int. J. Mol. Sci..

[20] Broer S. (2008). Amino acid transport across mammalian intestinal and renal epithelia. Physiol. Rev..

[21] Aretz I., Hardt C., Wittig I., Meierhofer D. (2016). An impaired respiratory electron chain triggers down-regulation of the energy metabolism and de-ubiquitination of solute carrier amino acid transporters. Mol. Cell. Proteom..

[22] Schlessinger A., Khuri N., Giacomini K.M., Sali A. (2013). Molecular modeling and ligand docking for solute carrier (SLC) transporters. Curr. Top. Med. Chem..

[23] Esslinger C.S., Cybulski K.A., Rhoderick J.F. (2005). N_γ_-Aryl glutamine analogues as probes of the ASCT2 neutral amino acid transporter binding site. Bioorg. Med. Chem..

[24] Chiu M., Sabino C., Allegri M., Bianchi M.G., Bussolati O. (2016). Multiple glutamine transporters sustain the growth of glutamine synthetase-negative human oligodedroglioma cells. FASEB J..

[25] Udeshi N.D., Mani D.R., Eisenhaure T., Mertins P., Jaffe J.D., Clauser K.R., Hacohen N., Carr S.A. (2012). Methods for quantification of in vivo changes in protein ubiquitination following proteasome and deubiquitinase inhibition. Mol. Cell. Proteom..

[26] Amann T., Hellerbrand C. (2009). GLUT1 as a therapeutic target in hepatocellular carcinoma. Expert Opin. Ther. Targets.

[27] Pinheiro C., Longatto-Filho A., Azevedo-Silva J., Casal M., Schmitt F.C., Baltazar F. (2012). Role of monocarboxylate transporters in human cancers: State of the art. J. Bioenerg. Biomembr..

[28] King M.P., Attardi G. (1996). Isolation of human cell lines lacking mitochondrial DNA. Methods Enzymol..

[29] Meierhofer D., Halbach M., Sen N.E., Gispert S., Auburger G. (2016). *Atxn2*-knock-out mice show branched chain amino acids and fatty acids pathway alterations. Mol. Cell. Proteomics.

[30] Gielisch I., Meierhofer D. (2015). Metabolome and proteome profiling of complex I deficiency induced by rotenone. J. Proteome Res..

[31] Tyanova S., Temu T., Sinitcyn P., Carlson A., Hein M.Y., Geiger T., Mann M., Cox J. (2016). The perseus computational platform for comprehensive analysis of (prote)omics data. Nat. Methods.

