# Supplementary material for: ρ0 Cells Feature De-Ubiquitination of SLC Transporters and Increased Levels and Fluxes of Amino Acids
Source: Int J Mol Sci. 2017 Apr 20;18(4):879. doi: 10.3390/ijms18040879 (PMC5412460; doi:10.3390/ijms18040879)
Supplement: Supplementary file 1 [file ijms-18-00879-s001.zip › Supporting tables content.pdf]

De-ubiquitination of SLC transporters leads to increased levels and fluxes of amino acids in human osteosarcoma ρ^0 cells

Andre Medina[1], Marcin Banaszczak[1,2], Yang Ni[1], Ina Aretz[1], and David Meierhofer[1]*

Supplementary Tables. This material is available free of charge via the Internet at http://pubs.acs.org.

Supplementary Table S1: De-ubiquitination of SLC transporters in ρ0 versus 143B.TK- cells. SLC transporters, including the specific site of ubiquitination are indicated with mean fold changes, 1/fold changes and SD.

Supplementary Table S2: Integrated peak areas in counts per second (cps) for all six biological replicates (A-F) for each detected amino acid in ρ^0 and 143B.TK$^-$ cells at time points 2.5, 5, 10, and 20 minutes. Data are normalized for protein content and internal standard, n=6.

Supplementary Table S3: Concentration of amino acids in $^{13}C^{15}N$ and $^{12}C^{14}N$ labeled cell culture media.

Supplementary Table S4: Integrated peak areas in counts per second (cps) for all three biological replicates (A-C) for each detected $^{13}C^{15}N$ labeled amino acid in ρ^0 and 143B.TK$^-$ cells at time points 0 (control without GPNA) and after 10 minutes incubation with the SLC inhibitor GPNA. Data are normalized for protein content and internal standard, n=3.

Supplementary Table S5: List of all amino acids and their $^{13}C^{15}N$ labeled forms with transitions, MS conditions and MRM ion ratios.
